# Anosognosia in Dementia: Evaluation of Perfusion Correlates Using 99mTc-HMPAO SPECT and Automated Brodmann Areas Analysis

**DOI:** 10.3390/diagnostics12051136

**Published:** 2022-05-04

**Authors:** Varvara Valotassiou, Nikolaos Sifakis, Chara Tzavara, Evi Lykou, Niki Tsinia, Vasiliki Kamtsadeli, Dimitra Sali, George Angelidis, Dimitrios Psimadas, Eudoxia Theodorou, Ioannis Tsougos, Sokratis G. Papageorgiou, Panagiotis Georgoulias, John Papatriantafyllou

**Affiliations:** 1 Nuclear Medicine Department, University Hospital of Larissa, 41110 Larissa, Greece; htzavara@med.uoa.gr (C.T.); angelidis@protonmail.ch (G.A.); dpsimad@chem.uoa.gr (D.P.); evitheodorou@yahoo.gr (E.T.); georgoulias@hotmail.com (P.G.); 2 Nuclear Medicine Department, Faculty of Medicine, University of Thessaly, Viopolis, 41500 Larissa, Greece; 3 Nuclear Medicine Department, “Alexandra” General Hospital, 11528 Athens, Greece; nik.osif@hotmail.com; 4 3rd Age Day Care Center, IASIS, 16562 Athens, Greece; evilykou@gmail.com (E.L.); vkamtsadeli@gmail.com (V.K.); jpapatriantafyllou@gmail.com (J.P.); 5 1st University Psychiatric Department, Aeginition Hospital, 11528 Athens, Greece; ntsinia@gmail.com; 6 Neurology Department, Evrokliniki, 11521 Athens, Greece; mimitel2003@yahoo.com; 7 Medical Physics Department, Faculty of Medicine, University of Thessaly, Viopolis, 41500 Larissa, Greece; tsougos@med.uth.gr; 8 2nd University Department of Neurology, Attikon Hospital, 12462 Athens, Greece; sokpapa@med.uoa.gr; 9 Memory Disorders Clinic, Medical Center, 15125 Athens, Greece

**Keywords:** anosognosia, dementia, brain perfusion, SPECT, Brodmann areas

## Abstract

(1) Background: Considerable inconsistency exists regarding the neural substrates of anosognosia in dementia in previous neuroimaging studies. The purpose of this study was the evaluation of anosognosia perfusion correlates across various types of dementia using automated Brodmann areas (BAs) analysis and comparison with a database of normal subjects. (2) Methods: We studied 72 patients: 32 with Alzheimer’s disease, 26 with frontotemporal dementia—FTD (12 behavioral FTD, 9 semantic FTD, 5 Progressive Non-Fluent Aphasia), 11 with corticobasal syndrome, and 3 with progressive supranuclear palsy. Addenbrook’s Cognitive Examination—Revised (ACE-R) mean(±SD) was 55.6(±22.8). For anosognosia measurement, the Anosognosia Questionnaire—Dementia was used. Total anosognosia score mean(±SD) was 22.1(±17.9), cognitive anosognosia score mean(±SD) was 18.1(±15.1) and behavioral–mood anosognosia score mean(±SD) was 3.3(±4.7). (3) Results: Higher anosognosia total score was associated with hypoperfusion in the inferior temporal, anterior cingulate, and inferior frontal cortices of the right hemisphere (BAs 20R, 24R, 32R, 45R). Higher anosognosia cognitive score was correlated with hypoperfusion in the left middle and anterior temporal cortices, and right dorsal anterior cingulate cortex (BAs 21L, 22L, 32R). No association was found with behavioral–mood anosognosia. (4) Conclusions: Automated analysis of brain perfusion Single Photon Emission Computed Tomography could be useful for the investigation of anosognosia neural correlates in dementia.

## 1. Introduction

Anosognosia is a term used across a wide range of neurological clinical cases and refers to the unawareness of the presence of a disorder and its associated deficits, such as cognitive and behavioral impairments [1,2,3,4,5]. The symptoms have also been described as loss of insight, impaired self-awareness, or self-consciousness [1]. Anosognosia was described for the first time in 1914 by Joseph Babinski in stroke patients who appeared unawareness of limb weakness or paralysis and progressed to dementia after some time [6].

Anosognosia has been reported in various forms of degenerative dementia [7,8,9]. Demented patients are frequently unaware of their cognitive and behavioral impairments and underestimate their deficits in multiple domains, despite facing major changes in their lives as a result of cognitive and socioemotional deterioration [10]. Anosognosia in dementia is a major problem, with significant consequences for patients and their caregivers, because it may delay medical evaluation and treatment, expose patients to risky behaviors, cause accidents, and worsen the prognosis, as well as increasing the caregivers’ burden [11,12].

The incidence and prevalence of anosognosia vary greatly across dementia types [13]. In Alzheimer’s disease (AD), the symptom may be observed in the early stages, even in the mild cognitive impairment stage [14,15]. The incidence ranges from 21% to 38.3% and the prevalence from 24% to 80% and increases as the disease worsens, affecting awareness of memory deficits in parallel with behavioral alterations [16,17,18,19,20]. Among the syndromes of the frontotemporal lobar degeneration (FTLD) spectrum, anosognosia has been reported mainly in the behavioral variant of frontotemporal dementia (bv-FTD) [7,21,22] and targeted specific domains such as personality [21], language, executive functions, and behavioral disturbances [22]. In the remaining syndromes of the FTLD spectrum, there are limited data regarding the frequency and characteristics of anosognosia [3,23].

The neuroanatomical correlates of anosognosia are very imprecise and an improved exploration is needed to shed light on the underlying mechanisms, resulting in interventions that would lessen the impact of the symptom on well-being. Previous nuclear neuroimaging studies have assessed anosognosia mainly in AD and bvFTD [15,24,25], examining only limited brain regions using the region of interest (ROI) approach [15,25].

The aim of this study was to evaluate perfusion correlates of anosognosia in demented patients with various forms of dementia using brain Single Photon Emission Computed Tomography (SPECT) and automated analysis of Brodmann areas (BAs) perfusion after comparison with a normal subjects database. We included in the analysis the whole brain cortex and not arbitrary selected regions used in the ROI method, avoiding the possibility of overlooking significant areas associated with anosognosia. Changes in perfusion in specific BAs may provide a further understanding of anosognosia in dementia of various etiologies.

## 2. Materials and Methods

### 2.1. Patients

Patients from an outpatient Memory Clinic were prospectively studied. We used the Diagnostic and Statistical Manual IV (DSM-IV) criteria for the clinical diagnosis of dementia [26], the National Institute of Neurologic and Communicative Disorders and Stroke, and the AD and Related Disorders Association Work Group (NINCDS-ADRDA) criteria for the diagnosis of AD [27], the Neary criteria for the diagnosis of FTD [28], the Bak and Hodges criteria for the diagnosis of Corticobasal Syndrome (CBS) [29], and the National Institute of Neurological Disorders and Stroke and Society for Progressive Supranuclear Palsy-PSP (NINDS-SPSP) criteria for the diagnosis of PSP [30].

All the patients underwent a neuropsychological evaluation with a battery of tests, including the Mini Mental State Examination (MMSE) for dementia rating and Addenbrook’s Cognitive Examination–Revised (ACE-R). The Anosognosia Questionnaire—Dementia (AQ-D) was used for anosognosia measurement [31]. The AQ-D is a 30-item questionnaire, and it is divided into two sections for the assessment of awareness of both cognitive deficits and behavioral–mood changes. Each answer is rated as follows: never (0 points), sometimes (1 point), usually (2 points), and always (3 points). According to this instrument, higher scores correspond to more severe anosognosia. It is administered to patients and caregivers, and the discrepancy between the scores (caregiver score–patient score) is defined as the anosognosia score. Additionally, all the patients underwent Computed tomography (CT) or Magnetic Resonance Imaging (MRI) to exclude vascular or structural brain lesions. Patients with psychiatric or other neurological disorders, based on their clinical history or the information received from their families or caregivers and the neuropsychological tests, as well as pregnant women, were excluded from the study.

Informed consent was obtained from all patients or their caregivers before the study according to the Hospital Ethics Committee guidelines based on the ethical guidelines of the Helsinki Declaration of 1975, as revised in 2000. All patients and caregivers received written directions on radioprotection before the study.

### 2.2. SPECT Studies

All the patients underwent a brain SPECT 20 min after the intravenous administration of 740 MBq of 99mTechnetium (99mTc) hexamethyl propylene amine oxime (HMPAO-Ceretec, Nycomed Amersham Sorin S.R.L., GE Healthcare Amersham Health) on a dual-head gamma camera (ADAC Forte) equipped with low-energy ultra-high resolution parallel-hole collimators. Acquisition parameters involved step-and-shoot mode (128 projections, 35 s/projection), 128 × 128 matrix, and photopeak centered at 140 keV with a symmetrical 10% window. We used the filtered back-projection technique for reconstruction and a Generic Wiener filter for smoothing.

On the reconstructed data, we applied the NeuroGamTM software (SegamiCorporation, Columbia, SC, USA www.segamicorp.com) with a predefined BA template for the automated comparison of perfusion in BAs of the left (L) and right (R) hemispheres in our patients’ group with BA perfusion of a database of normal subjects with the same age (provided by the software), as we have described in detail previously [32,33,34]. SPECT acquisition parameters in our study were the same as those used in the normal database subjects. Perfusion values in BAs are expressed as standard deviation (SD) differences from the age-matched normal subjects.

### 2.3. Statistical Analysis

Quantitative variables were expressed as mean values (standard deviation) and as median (interquantile range), while qualitative variables were expressed as absolute and relative frequencies. Spearman correlation coefficients (rho) were used to explore the association between anosognosia scales and BA perfusion. Via linear regression analysis, it was examined the association between anosognosia scales and BA perfusion after adjusting for gender, age, ACE-R, and years of education. Adjusted regression coefficients (β) with standard errors (SE) were computed from the results of the linear regression analyses. For cognitive and psychological anosognosia scales, logarithmic transformation was used, due to a lack of normality in their distribution. All reported *p* values are two-tailed. Statistical significance was set at *p* < 0.05 and analyses were conducted using IBM SPSS statistical software (version 22.0), New York, NY, USA.

## 3. Results

The sample consisted of 72 patients (66.7% females) with mean age 68.0 years (SD = 9.7 years). Their characteristics are presented in Table 1. Mean years of education were 10.5 (SD = 4.7 years). The majority of the patients (44.4%) were diagnosed with AD and mean years from disease onset were 3.2 (SD = 1.9 years). Mean ACE-R was 55.6 (SD = 22.8) and mean MMSE was 19.1 (SD = 7.3). Total anosognosia score ranged from −15 to 67, with mean value 22.1 (SD = 17.9); cognitive anosognosia score ranged from −18 to 65, with mean value 18.1 (SD = 15.1), and behavioral–mood anosognosia score ranged from −5 to 18, with mean value 3.3 (SD = 4.7) (Table 2).

A higher total anosognosia score was significantly associated with hypoperfusion in the right inferior temporal cortex (BA 20R) (r = −0.24; *p* = 0.041), left middle temporal cortex (BA 21L) (r = −0.29; *p* = 0.015), left anterior temporal cortex (BA 22L) (r = −0.29; *p* = 0.014), left ventral anterior cingulate cortex (BA 24L) (r = −0.27; *p* = 0.021), right ventral anterior cingulate cortex (BA 24R) (r = −0.26; *p* = 0.027), right dorsal anterior cingulate cortex (BA 32R) (r = −0.25; *p* = 0.036), left fusiform gyrus (BA 37L) (r = −0.24; *p* = 0.040), left inferior frontal gyrus—triangular part(BA 45L) (r = −0.26; *p* = 0.027), right inferior frontal gyrus—triangular part (BA 45R) (r = −0.24; *p* = 0.040), and left dorsolateral prefrontal cortex (BA 46L) (r = −0.25; *p* = 0.033) (Table 3). Moreover, a higher anosognosia cognitive score was significantly associated with hypoperfusion in the left dorsolateral prefrontal cortex (BA 9L) (r = −0.35; *p* = 0.003), rightdorsolateral prefrontal cortex (BA 9R) (r = −0.26; *p* = 0.028), left anterior prefrontal cortex (BA 10L) (r = −0.26; *p* = 0.029), right inferior temporal cortex (BA 20R) (r = −0.25; *p =* 0.039), left middle temporal cortex (BA 21L) (r = −0.30; *p =* 0.012), right middle temporal cortex (BA 21R) (r = −0.24; *p =* 0.048), left anterior temporal cortex (BA 22L) (r = −0.34; *p =* 0.004), left ventral anterior cingulate cortex (BA 24L) (r = −0.31; *p =* 0.009), right ventral anterior cingulate cortex (BA 24R) (r = −0.27; *p =* 0.021), left dorsal anterior cingulate cortex (BA 32L) (r = −0.29; *p =* 0.016), right dorsal anterior cingulate cortex (BA 32R) (r = −0.30; *p =* 0.010), left fusiform gyrus (BA 37L) (r = −0.23; *p =* 0.050), left angular gyrus (BA 39L) (r = −0.23; *p =* 0.050), right inferior frontal gyrus—opercular part (BA 44R) (r = −0.31; *p =* 0.008), left inferior frontal gyrus—triangular part (BA 45L) (r = −0.30; *p =* 0.010), right inferior frontal gyrus—triangular part(BA 45R) (r = −0.33; *p =* 0.005), left dorsolateral prefrontal cortex (BA 46L) (r = −0.33; *p =* 0.004), right dorsolateral prefrontal cortex (BA 46R) (r = −0.31; *p =* 0.009), orbital part of left inferior frontal gyrus (BA 47L) (r = −0.27; *p =* 0.021), and orbital part of right inferior frontal gyrus (BA 47R) (r = −0.28; *p =* 0.019). We did not find a significant correlation of anosognosia behavioral–mood score with hypoperfusion in any BA.

**Figure 1:**
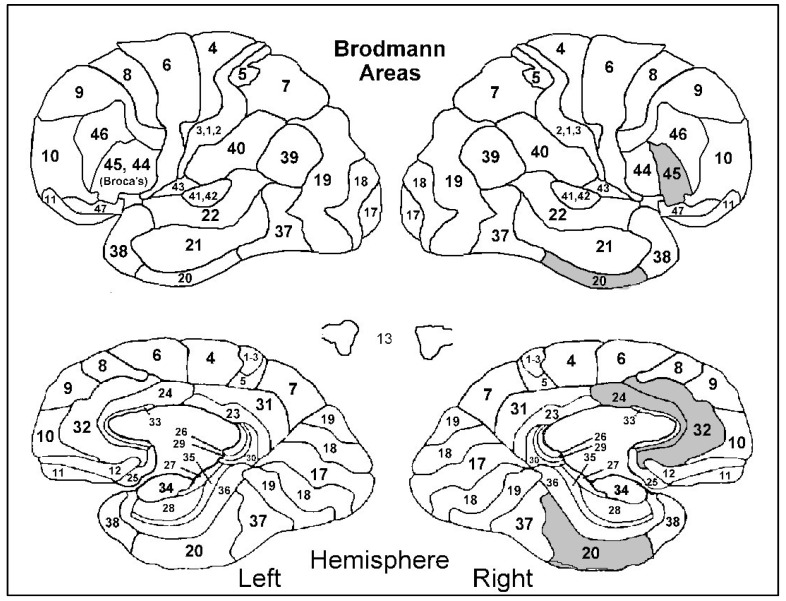
Hypoperfusion in Brodmann areas 20R, 24R, 32R, 45R (right inferior temporal cortex, right ventral and dorsal anterior cingulate cortex, and right inferior frontal gyrus—triangular part) was associated with a higher anosognosia total score.

**Figure 2:**
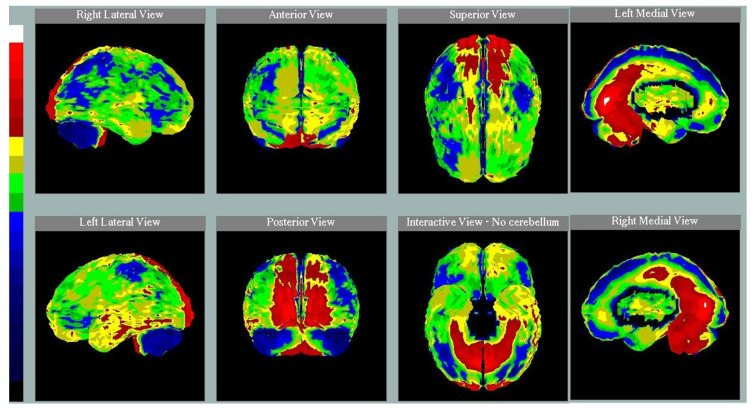
Brain perfusion SPECT with 99mTc-HMPAO in a 55-year-old woman with behavioral variant of frontotemporal dementia, Addenbrook’s Cognitive Examination—Revised score 67, 6 years of education, and total anosognosia score 29, showing hypoperfusion in inferior temporal cortex, anterior cingulate cortex, and inferior frontal gyrus, mainly of the right hemisphere.

**Figure 3:**
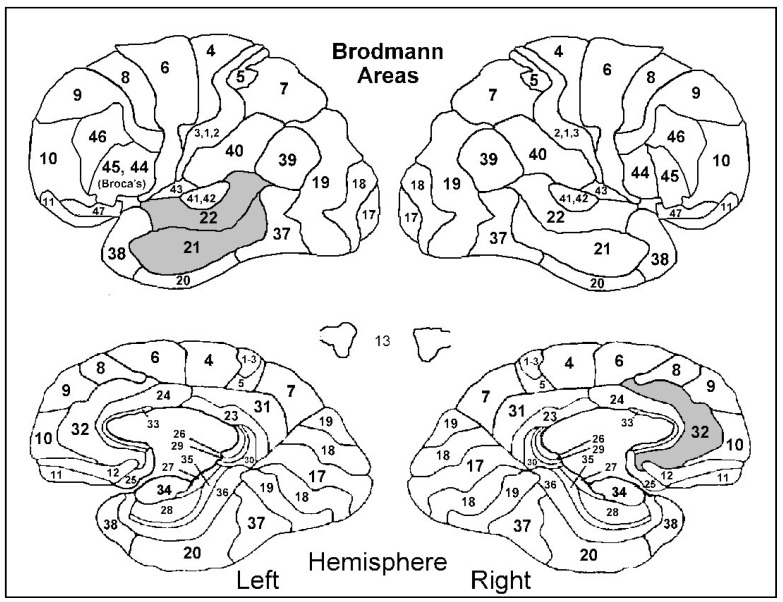
Hypoperfusion in Brodmann areas 21L, 22L, 32R (left middle and anterior temporal cortices and right dorsal anterior cingulate cortex) was significantly associated with a higher anosognosia cognitive score.

After adjusting for gender, age, ACE-R, and years of education, it was found that a higher anosognosia total score was associated with hypoperfusion in the right inferior temporal cortex (BA 20R) (β = −3.78; SE = 1.92; *p =* 0.041), right ventral anterior cingulate cortex (BA 24R) (β = −5.86; SE = 2.72; *p =* 0.030), right dorsal anterior cingulate cortex (BA 32R) (β = −3.91; SE = 1.90; *p =* 0.029), and right inferior frontal gyrus–triangular part (BA 45R) (β = −2.83; SE = 1.40; *p =* 0.026) (Figure 1 and Figure 2). Furthermore, a higher anosognosia cognitive score was significantly associated with hypoperfusion in the left middle temporal cortex (BA 21L) (β = −0.07; SE = 0.03; *p =* 0.035), left anterior temporal cortex (BA 22L) (β = −0.07; SE = 0.03; *p =* 0.039), and right dorsal anterior cingulate cortex (BA 32R) (β = −0.07; SE = 0.03; *p =* 0.042) (Figure 3).

## 4. Discussion

Anosognosia in dementia is a highly disruptive symptom and the exploration of its neuroanatomical correlates is of particular interest. The purpose of this study was the evaluation of the perfusion correlates of anosognosia in a population with various types of neurodegenerative dementias to reveal specific BAs implicated in the symptom across disparate dementia etiologies. Moreover, we examined the whole brain cortex using a voxel-based method to avoid overlooking certain regions.

We found that higher anosognosia total scores were correlated with hypoperfusion in the frontal and temporal areas, as well as in the bilateral (but mainly on the right) anterior cingulate. Moreover, higher anosognosia cognitive scores were significantly associated with hypoperfusion in the frontal and temporal regions, in the bilateral anterior cingulate, and in the angular gyrus of the left parietal lobe. However, after adjusting for gender, age, ACE-R, and years of education, we found that a higher anosognosia total score was associated with hypoperfusion in the right inferior frontal gyrus—triangular part (BA 45R), right anterior (ventral and dorsal) cingulate cortex (BA 24R, 32R), and right inferior temporal cortex (BA 20R), while a higher anosognosia cognitive score was significantly associated with hypoperfusion in the right dorsal anterior cingulate cortex (BA 32R), as well as in the left middle and anterior temporal cortex (BA 21L, 22L). Our findings are, in general, in concordance with the findings of other studies, although there is great heterogeneity in the literature concerning patient selection and the specific type of dementia studied, since most studies were performed in AD and bvFTD, as well as anosognosia assessment instruments, neuroimaging techniques, and SPECT quantification methods. The previous factors may have influenced the findings in various studies and may account for the discrepancies between them and our study.

In AD, anosognosia was found to correlate with decreased perfusion in frontal areas [35,36,37,38,39] such as the left orbitofrontal cortex and the correlated regions extended to the right orbitofrontal cortex [24], the right dorsolateral frontal cortex [25], and the right inferior frontal gyrus [15], as well as in the anterior cingulate [35]. Brain glucose metabolism with 18F-fluorodeoxyglucose (FDG) Positron Emission Tomography (PET) studies showed dysfunction of the cortical midline areas [5,14,40,41,42] in the early stages of AD and involvement of the frontal cortex in the later stages [43,44,45]. This pattern parallels the histological changes as described by Braak and Braak [46], according to whom the mediotemporal cortex is initially affected, followed by the posterolateral cortical areas and extension to the frontal cortex at later stages [47]. Additionally, grey matter volumes in MRI studies have been reported to associate significantly with the presence of anosognosia. More specifically, the majority of these studies highlight an association between the symptom and reduced volumes in the prefrontal cortex [48,49,50,51], cingulate cortex [35,52], medial temporal lobe [50,52,53], subcortical structures [51], and cerebellum [52]. In FTD, anosognosia was found to correlate mainly with either hypoperfusion, hypometabolism, or atrophy in the right frontal lobe [2,51,54,55], in bilateral temporal poles [4], or in an area posterior to the right superior temporal sulcus [56]. However, the main research has been conducted in bvFTD, while limited data are available regarding the remaining neurodegenerative disorders that share pathological and genetic features with FTD, such as the CBS or the PSP [3,23]. Muñoz-Neira and his colleagues recently reviewed the neuroimaging neural correlates of impaired insight in various forms of FTD [57]. They reported a correlation that varies according to the object of insight in FTD syndromes. More specifically, impaired insight of the presence of disease or diagnosis or health condition was associated with hypometabolism or hypoperfusion in the right frontal cortex [2,58] or atrophy of frontal areas involving the left orbitofrontal cortex and the right anterior cingulate [59]. Additionally, altered insight into social cognition correlated with grey matter atrophy in the right inferotemporal regions [60], and altered insight for memory was correlated with the frontal and parietal lobes and limbic structures [61].

Several theoretical models have been proposed for the pathogenesis of anosognosia in degenerative dementias and mainly in AD, and previous neuroimaging studies, as well ours, are generally in alignment with these models [62]. It is considered that specific prefrontal areas, such asthe medial prefrontal cortex and the anterior cingulate, may have a pivotal role in the executive system and impaired connections within the system may result in executive anosognosia [63,64,65]. It is also considered that memory or executive anosognosia is associated with the degeneration of frontotemporal networks, which are believed to be responsible for the integrity of the cognitive awareness system [66]. In addition, anosognosia has been linked to the temporoparietal cortex since impairment in this area can result in a variety of disorders associated with knowledge of body and perception [67]. Other researchers assume that the pathological substrates of memory anosognosia are located in regions responsible for autobiographical conceptual memory, such asthe medial temporal lobe [53,68]. Moreover, in functional neuroimaging studies, it has been shown that the temporoparietal regions (especially on the right) are activated when subjects are asked to distinguish themselves from others’ attributes [4,69], which means that this brain region may be part of the network that encloses the representation of the self and has a role for self-awareness [70].

In FTD, the main research has been focused on bvFTD, and it has been reported that disease unawareness correlates with the right frontal cortex, while altered insight into social cognition correlates with the frontal areas, as well as with the temporal gyrus, insula, parahippocampus, and amygdala, and impaired insight into memory problems seems to be related tothe frontal cortex, postcentral gyrus, parietal cortex, and posterior cingulate [57]. Interestingly, MRI studies in psychiatric diseases have shown that poor awareness of illness is associated with grey matter thinning in the left middle frontal and inferior temporal cortices, suggesting that the neural correlates of insight involve a network of brain structures, which are located not only in the frontal lobes but in the parietal and temporal lobes, too [71,72].

Concerning the hemispheric predominance of anosognosia neural correlates, it has been reported a correlation of global anosognosia for cognitive impairment with the right hemisphere in AD, as well as in hemiplegic patients due to stroke or brain injury [15,24,25,73,74,75]. In these studies, the symptom was associated with hypoperfusion in the prefrontal, temporoparietal, and temporo-occipital regions. However, in bvFTD patients, anosognosia has been found to correlate with hypoperfusion in the left temporal pole [4] and the right frontal regions [2]. It is considered that the right lateralization of findings in previous studies associated with anosognosia, especially in FTD, could be attributed to the existence of a specific association between lack of insight and impairment of the right frontal lobe, which dominates emotions [2,76,77,78]. In our study, we also found a correlation of total anosognosia score with the right frontal, temporal, and cingulate cortices. We also found that cognitive anosognosia was correlated with hypoperfusion in the left temporal cortex and right anterior cingulate. Cocchini et al. [79] reported that the frequency of anosognosia in left-hemisphere brain damages may have been underestimated and that anosognosia at least for motor impairment may also be associated with left-hemispheric lesions.

In our study, we did not find a significant correlation between anosognosia behavioral–mood score and hypoperfusion in any BA. It would be supposed with caution that these specific features in the AQ-D instrument share completely different brain regions across various dementia types. In concordance with the previous opinion, it has been considered that insight into specific neuropsychological/behavioral domains is sustained by specific brain regions [57]. To our knowledge, there are no published studies dealing with behavioral–mood anosognosia and neuroimaging, or the existing published studies investigated the neural substrates in specific objects such as memory and executive function. Additionally, there is significant diversity regarding anosognosia evaluation methods in the literature, as well as high complexity of the term and a lack of consensus about its definition [57].

## 5. Potential limitations

A potential limitation of our study is that anosognosia BA perfusion correlates were not tested separately in the different dementia types. We did not present results from this analysis because the number of patients and the statistical power per dementia type were not the same (and in some cases was very small) and could lead to a biased conclusion. For this reason, we analyzed dementia types altogether and made adjustments for ACE-R.

## 6. Conclusions

The findings of this study suggest an association between anosognosia and decreased perfusion in the frontal, temporal, and anterior cingulate cortices. Brain perfusion SPECT with automated BA analysis and comparison with a normal database would serve as a neuroimaging biomarker to provide insight into the mechanism of anosognosia in dementia, as well as in other neurological diseases. The understanding of anosognosia neural substrates is of great clinical interest and would aid in the disease prognostication.

## Figures and Tables

**Table 1:** Sample characteristics.

	N (%)
Gender	
Males	24 (33.3)
Females	48 (66.7)
Age, mean (SD)	68 (9.7)
Years of education, mean (SD)	10.5 (4.7)
Diagnosis	
AD	32 (44.4)
bvFTD	12 (16.7)
PNFA	5 (6.9)
svFTD	9 (12.5)
CBS	11 (15.3)
PSP	3 (4.2)
Dextrality	
Right	71 (98.6)
Left	1 (1.4)
Years from disease onset, mean (SD)	3.2 (1.9)
MMSE, mean (SD)	19.1 (7.3)
ACE-R, mean (SD)	55.6 (22.8)
Total anosognosia, mean (SD)	22.1 (17.9)
median (IQR)	20 (8–34.5)
Cognitive anosognosia, mean (SD)	18.1 (15.1)
median (IQR)	15 (9–29)
Behavioral–mood anosognosia, mean (SD)	3.3 (4.7)
median (IQR)	3 (0–6)

AD Alzheimer’s disease, FTD frontotemporal dementia, bvFTD behavioral variant FTD, PNFA Progressive Non-Fluent Aphasia, svFTD semantic variant FTD, CBS corticobasalsyndrome, PSP progressive supranuclear palsy, MMSE Mini Mental State Examination, ACE-R Addenbrook’s Cognitive Examination—Revised, SD standard deviation, IQR interquantilerange.

**Table 2:** Descriptive statistics for the anosognosia dimensions.

	Mean (SD)	Median (IQR)
Total anosognosia	22.1 (17.9)	20 (8–34.5)
Cognitive anosognosia	18.1 (15.1)	15 (9–29)
Behavioral–mood anosognosia	3.3 (4.7)	3 (0–6)

**Table 3:** Spearman’s correlation coefficients of anosognosia scores with BA perfusion after comparison with normal database.

BAs	Total Anosognosia	Cognitive Anosognosia	Behavioral–Mood Anosognosia
123L	0.05	−0.03	0.19
123R	0.00	−0.08	0.10
4L	0.16	0.08	0.22
4R	0.16	0.09	0.16
5L	0.10	0.01	0.14
5R	0.05	−0.03	0.14
6L	0.11	0.02	0.17
6R	0.12	0.06	0.09
7L	−0.03	−0.11	0.16
7R	−0.03	−0.10	0.06
8L	−0.09	−0.22	0.09
8R	−0.10	−0.20	0.04
9L	−0.23	−0.35 **	−0.01
9R	−0.16	−0.26 *	−0.02
10L	−0.16	−0.26 *	−0.03
10R	−0.09	−0.18	−0.01
11L	−0.01	−0.05	−0.05
11R	0.00	−0.03	−0.04
12L	0.05	0.01	−0.04
12R	0.03	0.01	−0.05
17L	0.04	−0.04	0.11
17R	0.17	0.08	0.16
18L	−0.01	−0.09	0.06
18R	0.11	0.01	0.16
19L	−0.09	−0.16	0.05
19R	0.05	−0.02	0.09
20L	−0.19	−0.19	−0.05
20R	−0.24 *	−0.25 *	−0.13
21L	−0.29 *	−0.30 *	−0.05
21R	−0.21	−0.24 *	−0.06
22L	−0.29 *	−0.34 **	−0.04
22R	−0.13	−0.18	−0.09
23L	−0.17	−0.20	−0.03
23R	−0.09	−0.16	0.04
24L	−0.27 *	−0.31 **	−0.11
24R	−0.26 *	−0.27 *	−0.07
25L	0.00	−0.04	0.01
25R	0.08	0.05	0.11
28L	−0.05	−0.05	−0.03
28R	−0.20	−0.19	−0.11
31L	−0.01	−0.08	0.14
31R	0.05	−0.02	0.14
32L	−0.22	−0.29 *	−0.07
32R	−0.25 *	−0.30 *	−0.10
36L	−0.14	−0.13	0.03
36R	−0.20	−0.20	−0.07
37L	−0.24 *	−0.23 *	0.01
37R	−0.20	−0.20	−0.06
38L	−0.13	−0.16	0.00
38R	−0.14	−0.18	−0.08
39L	−0.22	−0.23 *	0.03
39R	−0.16	−0.17	−0.10
40L	−0.21	−0.23	0.01
40R	−0.13	−0.15	−0.06
44L	−0.14	−0.23	−0.04
44R	−0.22	−0.31 **	−0.06
45L	−0.26 *	−0.30 **	−0.12
45R	−0.24 *	−0.33 **	−0.13
46L	−0.25 *	−0.33 **	−0.10
46R	−0.21	−0.31 **	−0.07
47L	−0.20	−0.27 *	−0.14
47R	−0.19	−0.28 *	−0.07

* *p* < 0.05; ** *p* < 0.01; BAs: Brodmann areas, L: left, R: right.

## Data Availability

Data available on request due to privacy. The data presented in this study are available on request from the corresponding author.
